# Cross-Sectional Study Using Virtual Reality to Measure Cognition

**DOI:** 10.3389/fspor.2020.543676

**Published:** 2021-02-11

**Authors:** Yeonhak Jung, Jonathan B. Dingwell, Brett Baker, Preeti Chopra, Darla M. Castelli

**Affiliations:** ^1^Department of Kinesiology & Health Education, The University of Texas at Austin, Austin, TX, United States; ^2^Department of Curriculum & Instruction, The University of Texas at Austin, Austin, TX, United States; ^3^Department of Kinesiology, The Pennsylvania State University, State College, PA, United States

**Keywords:** dual-tasking, cognitive-motor interference, cognition, exercise, virtual reality, behavior response, exercise intensity, cognitive demand

## Abstract

Dual-task research is limited in its transferability to authentic contexts because laboratory conditions do not replicate real-world physical activity and decision-making scenarios. Creating valid, reliable methodologies to assess physiological and behavioral responses under varying physical and cognitive demands using virtual reality (VR) environment addresses this limitation. This study determined the feasibility of using VR to investigate the effects of dual-tasking on healthy young adults' cognitive performance. Three dual-tasking conditions (i.e., standing, preferred-paced walking, and fast-paced walking, each with blocked congruent and incongruent tasks) were developed. Using a within-subjects, randomized design, thirty-two young adults (17 female, mean age = 21.03 ± 2.86) were randomly assigned to a starting condition but experienced all three conditions. Physiological responses of heart rate (HR) and accelerometry data measured energy expenditure as the physical demand. Behavioral responses of reaction time and error rate quantified cognitive performance. Results indicated that (a) each condition verified independent physiological and behavioral responses; (b) reaction time and error rate during preferred walking or fast-paced walking dual-tasking conditions was significantly lower than standing condition; and surprisingly, (c) congruent tasks showed lower reaction time than the incongruent tasks. These findings suggest that it is feasible to use VR to assess the effects of dual-task conditions. Specifically, walking can optimize the motor-cognitive dual-task performance, compared to standing. These findings may be attributed to the dose-response effects of exercise intensity. Future studies should incorporate advanced technology such as the VR exercise.

## Introduction

Technological advances have manifested a need for increased inhibitory control, given the number of stimuli experienced by humans in daily living. The intensified demand for information processing affects decision-making (Crone and Dahl, 2012) and cognitive reasoning (Houdé and Borst, 2014, 2015). Although people may think of themselves as such, humans are not multitaskers. Instead, humans shift attention between tasks while focusing on a primary stimulus and blocking out distractions that may emerge from secondary stimuli. For example, a driver needs to selectively attend to the Global Positioning System (GPS) information that simultaneously provides a visual map and verbal directions requiring a motor response. Humans uniquely process each piece of information first through the source of the stimuli (e.g., sight, sound, touch, and/or smell) and then by discriminately prioritizing the information they believe has the most significant relevance to their goal. Most decisions like the one just described are considered automatic information processing and therefore considered cold cognition (Leshem et al., 2020). Although some cognitive decisions are both complex and influenced by emotion, all of the conditions in this research study were classified as cold cognitive decisions. Further, given the minimal risk of performing each task, little emotion was involved.

### Background/Rationale

When humans are asked to perform two tasks simultaneously, the two tasks are executed interdependently. However, because they have distinct and separate goals (Sigman and Dehaene, 2008), it is called dual-tasking. The study of dual-tasking has been around for some time. With the emergence of virtual reality (VR) and an interest in the effects of acute exercise on cognitive performance, new paradigms and conceptual frameworks are beginning to emerge. Most studies currently reflect the traditional assumption that dual-tasking deteriorates the performance on one or both tasks because an individual has limited resources for cognitive processing (Kahneman, 1973). The underlying premise of dual-tasking studies is that resources are limited, and when they have to be shared between two tasks simultaneously, performance will degrade relative to when and how often each task is performed (Plummer and Eskes, 2015; Herold et al., 2018). Conversely, some studies have shown that dual-tasking that includes a motor task may benefit motor or cognitive performance (Altmann et al., 2015; Hazamy et al., 2017; Studer, 2018). For example, Altmann and colleagues found an improvement in motor performance during cognitive dual-tasks. Therefore, it is unclear how different types of exercise with varying physiological demands may influence cognitive performance, especially in real-world scenarios represented in VR environments.

Recently, the dual-tasking paradigm has been applied to understand the effects of texting and driving or walking. As previously mentioned, a driver must shift their attention between the two competing tasks. A pedestrian who uses a cell phone while walking is more susceptible to being involved in an accident because of delayed responses and increased variability of responses when navigating objects on the phone screen and within the walking space (Chopra et al., 2018). This relative change in performance associated with dual-tasking is referred to as *dual-task interference*. In a given moment, the required resources used to process information are available in fixed quantities. Thus, performance suffers when resources are exceeded by the demands of the task (Gopher and Navon, 1980). Given the competing demands for limited resources, conditions that elicit dual-task interference are generally used to assess cognitive abilities in the field of rehabilitation and gait studies.

There are additional plausible reasons for performance to deteriorate in dual-tasking conditions like walking and talking (e.g., Neider et al., 2011; Holtzer et al., 2014) or thinking while moving (Schaefer et al., 2015; Herold et al., 2018), which could be attributed to the motor-cognitive demands. Schaefer (2014) summarized three potential explanations for the dual-task interferences with different foci of interest. The first possible explanation is the *prioritization of walking* that the motor task is prioritized over cognition, because the motor task involves some threat to balance and risk of falling (Plummer and Eskes, 2015), such as prioritizing walking over talking on the phone (Verghese et al., 2007). The second potential explanation of dual-processing is *sensorimotor-cognitive interactions*. When comparing young and older adult participants in dual-tasking, older adults' limited resources led to increased walking instability, while young adults continue to show stable motor performance levels (Verrel et al., 2009; Schaefer, 2014). The underlying premise is that sensorimotor performance requires increased attentional resources with advanced age. Hence, most studies revealed that aging is the main contributing factor to gait balance, leading to cognitive decline under dual-task structure (Craik and Salthouse, 2011). The third explanation is related to the *beneficial effect of exercise on cognitive performance*. Even though most studies in this field assessed the cognitive benefits immediately after exercise, dual-tasking, like making decisions while playing a sport, may lead to the reallocation of attentional resources (Kamijo et al., 2009; Best, 2010; McMorris, 2015). As previously noted, various factors may influence dual-tasking situations, such as task difficulty, aging, arousal level, and postural threat. Despite these known effects, interference is often inadequately measured in dual-tasking studies, limiting what the researchers currently know about the effect of dual-task performance.

There are many dual-task studies focused on understanding the association between cognitive decision making and walking tasks (Al-Yahya et al., 2011; Schaefer, 2014); however, most of the studies have utilized a cognitive task that was not directly associated with the movement or with the real-world context (e.g., solve computational problems while walking). Further, the outcomes were indirectly extrapolated. No study to date has investigated the effects of cognitive tasks integrated into VR facilitated exercise conditions. Given the limitations of current methodologies, the direct effects of varying cognitive demands on motor performance remain unclear, and researchers still lack the knowledge and consistent application of cognitive tasks applicable to clinical and real-world settings. The present study attempts to address such identified gaps in the research by examining dual-task performance through direct measurement of behavioral and physiological responses during life-like conditions requiring differing cognitive demands. The researchers developed simulated real-world conditions that manipulated the difficulty of cognitive tasks and the amount of energy expended within each condition using an integrated VR environment and treadmill that mimics common everyday experiences (e.g., participant walks down a path and avoids or interacts with objects).

### Objectives

This study aimed to determine the feasibility of using VR to investigate the effects of dual-tasking on healthy young adults' cognitive performance. The research team developed three different VR conditions: (a) standing on a path and experiencing a block of congruent tasks followed by a block of incongruent tasks, (b) walking at a preferred speed down a path and experiencing a block of congruent tasks followed by a block of incongruent tasks, and (c) fast-paced walking down a path and experiencing a block of congruent tasks followed by a block of incongruent tasks. In accordance with previous dual-task research (Li et al., 2005), we hypothesized that participants would require more cognitive resources during walking and fast-paced walking than during a standing condition due to the prioritization of walking. Further, it was hypothesized that the incongruent task would require more attentional resources than congruent tasks, which would be reflected as slower reaction times and an increased frequency of error.

## Methods

### Study Design

**Figure 1:**
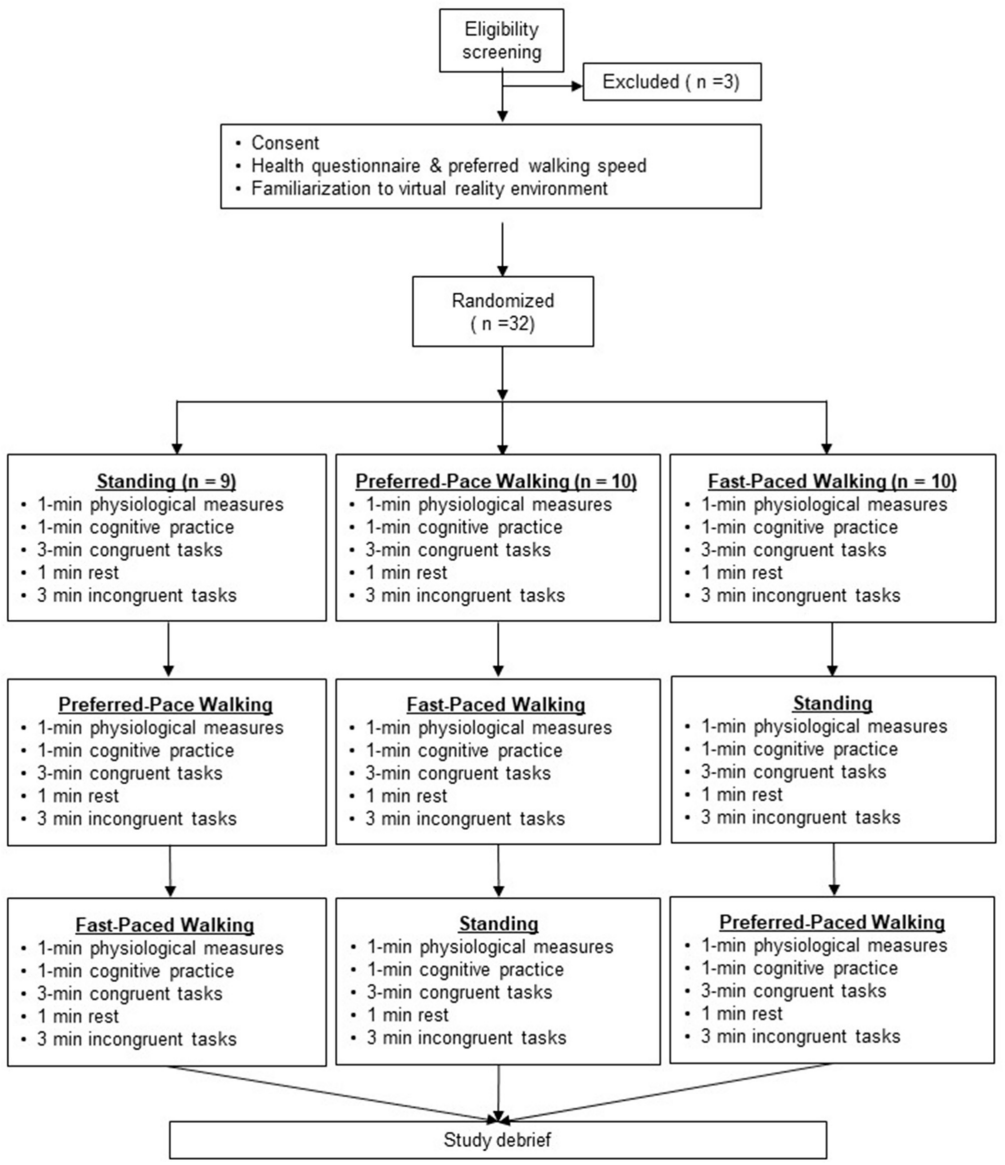
Summary of study protocol and randomization.

The study used a within-subject randomized design. Upon study approval by the Institutional Review Board at The University of Texas at Austin (IRB #2019-12-0107), potential participants were screened for eligibility. Those meeting the inclusion criteria came to the lab for one 90 min visit, completed a health screening questionnaire, baseline measures, and familiarization with the VR environment. Once comfortable with the VR, each participant experienced all three exercise conditions (Figure 1). During each condition, the researchers measured behavioral and physiological responses.

### Participants

Thirty-two self-reported healthy young adults (M age = 21.03 ± 2.86 years, 17 Females) participated (Table 1). Participants were recruited via flyers, online advertisements, and public announcements from central Texas. The participant's ages ranged from 19 to 29 years, and because each participant reported no adverse health characteristics, they were likely to have a level of physical fitness that would allow for an age-appropriate HR recovery between conditions. All participants were screened to ensure they had no history of falls, cardiovascular, neurological, or visual deficits that might have affected their walking ability. Physical health assessments, including HR, height, weight, and self-paced preferred walking speed (PWS), were measured before the participant was randomly assigned to the initial condition. Each participant received $20 for their participation.

**Table 1:** Participant Characteristics.

**Variable**	**Total (*n* = 29) M (SD)**	**Male (*n* = 15) M (SD)**	**Female (*n* = 14) M (SD)**
Age	21.24 (2.86)	22.33 (3.24)	20.07 (1.86)
Height (cm)	170.06 (8.47)	175.93 (6.46)	163.76 (5.18)
Body Mass (kg)	71.90 (13.80)	75.78 (13.83)	67.73 (12.95)
Body Mass Index (kg/m^2)^	24.78 (3.84)	24.39 (3.65)	25.19 (4.12)
HR resting (beat/min)	71.17 (15.14)	65.87 (12.72)	76.85 (15.89)
Preferred Walking Speed (m/s)	1.46 (0.18)	1.47 (0.22)	1.45 (0.13)

### Setting: Virtual Reality (VR) Environment

**Figure 2:**
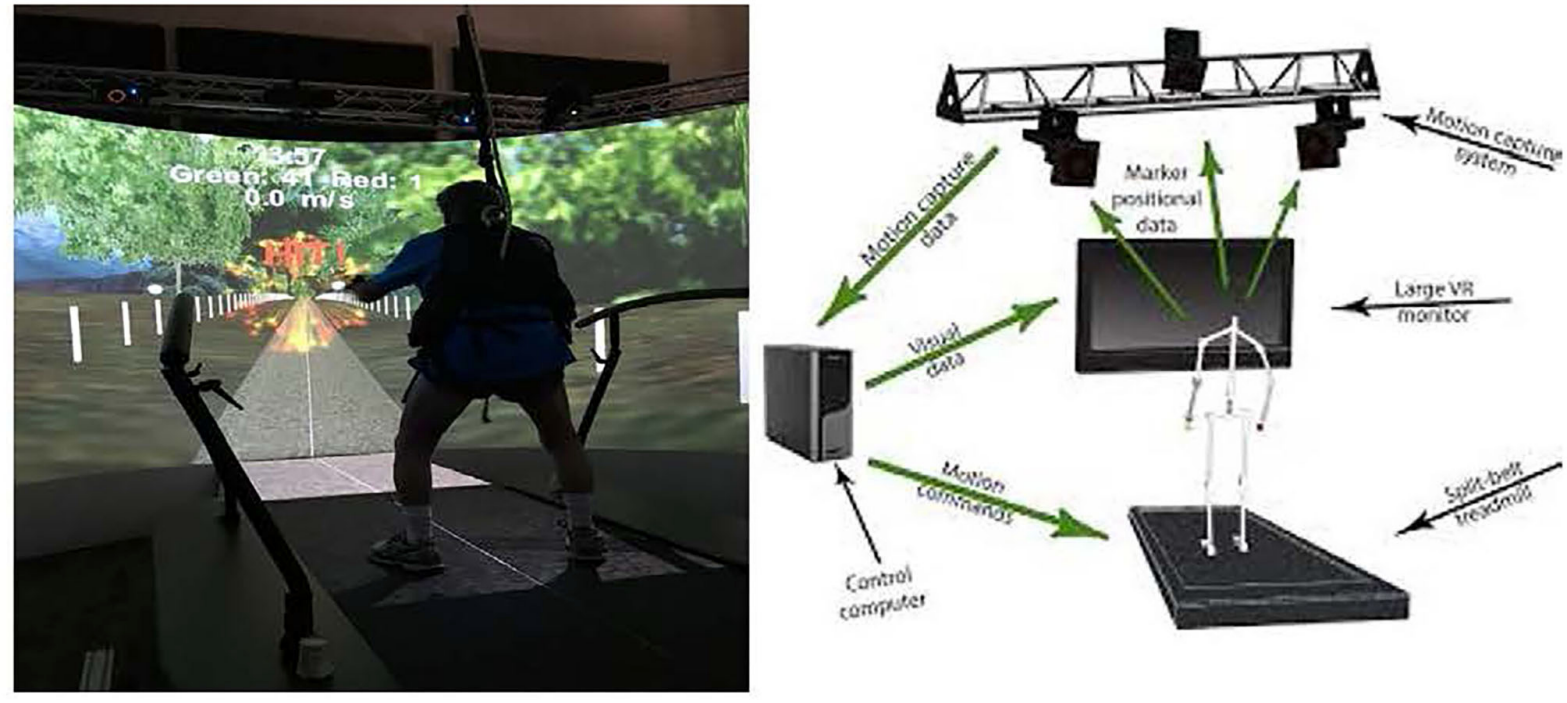
Visual and auditory feedback through VR screen during the dual-tasking condition.

The VR lab has a 1 m wide × 2 m, split-belt treadmill with an integrated VR projection screen to create authentic dual-tasking situations (Motekforce Link, Amsterdam, Netherlands). The VR scene was projected onto a 3-meter tall 180° degree semi-cylindrical screen in front of the treadmill (Figure 2). During the dual-tasking conditions, the screen displayed a computer-generated “trail road” environment through D-Flow software (Geijtenbeek et al., 2011), which realistically simulates the feel of being in the scenario. All participants wore a safety harness (Petzl, Crolles, and France) to prevent falls, and they were instructed to look straight ahead to the VR screen and avoid extraneous movement. Preferred walking speed (PWS) was measured before the dual-tasking condition. To assess the PWS and acclimate the participant to the VR, each participant walked for 5 min at various treadmill speeds. PWS was determined by starting from a relatively slow pace and slowly increasing the treadmill speed until the participant reported that the current speed felt “comfortable” without additional effort. This procedure was repeated three times and the average of the 3 “comfortable” speeds was taken as the participant's PWS.

### Motor-Cognitive Dual-Tasking

All participants experienced the three different VR dual-tasking conditions requiring the participant to avoid or interact with an object in their path: *standing congruent/incongruent; preferred-paced walking congruent/incongruent, and fast-paced walking congruent/incongruent*. The motor portion of the dual-task was exercise. Gait speed was used to manipulate exercise intensity in each exercise condition. Measures of heart rate (HR) and step counts confirmed the exercise intensity. The researchers established that low to moderate-intensity exercise corresponds to standing, whereas higher intensity exercise corresponds to fast-paced walking. During walking or fast-paced walking conditions, participants were instructed to walk at a PWS or 25% faster-walking speed than PWS on the VR treadmill, respectively.

The cognitive portion was based on two existing cognitive paradigms, the Go/No-Go and Oddball. The classic Go/No-Go paradigm (Gomez et al., 2007) examines response inhibition and response competition. In a typical Go/No go experiment, participants are instructed to respond by the keypress to the go stimulus and withhold responses to the No-Go stimulus (Ríos-Lago and Periáñez, 2010). Here, in this study, the participants are asked to interact with or avoid objects in the VR environment. The oddball paradigm (Picton, 1992) examines behavioral and neural responses to novel events. In this paradigm, an improbable series of unique and unexpected novel events are presented, in addition to targets and standards (Ríos-Lago and Periáñez, 2010). As an adaptation of this paradigm, a total of 60 colored target stimuli were displayed on the VR screen, with each object appearing on the screen for 3–5 s intervals. Additionally, visual and auditory feedback were given as each target was encountered.

The first block of 60 trials required the participant to interact with 80% of the objects, which was considered a congruent task. Once the block of congruent tasks was completed, the participant stood for 1 min to receive the opposite directions. The second set of directions required the participant to interact with the object they previously had to avoid (20%), thus reversing the expected behavioral response and introducing interference into the information processing. A congruent task was always introduced first, followed by the corresponding incongruent task for the same exercise mode and intensity to establish an expected behavioral response. The paired patterning of congruent and incongruent was necessary to introduce interference as the incongruency.

### Measuring Behavioral and Physiological Responses

Cognitive performance was the dependent variable and was quantified by measuring reaction time and error rate. Behavioral responses of the reaction time and the percentage of error were calculated during dual-tasking conditions: (a) mean reaction time of correct responses and (b) mean percentage of incorrect responses to the non-target stimuli and missed target stimuli. The target stimuli were presented for 3,000 ms. If the participant did not identify a correct response within 3 s, it was considered incorrect. Only the correct responses were used as data for the reaction time. Physiological responses of step counts and HR were tracked using an Actigraph GT3X accelerometer (Santos-Lozano et al., 2013) and a Polar HR monitor (Ceesay et al., 1989). In combination, these data provided a total volume and intensity of exercise, thus allowing us to compare the physiological effects of different conditions.

An integrated 10 camera Vicon motion capture system (Oxford Metrics, Oxford, UK) and D-Flow software recorded the participant's movement to project it on to the screen. The participants wore gloves with hand markers that captured reaction time and error rate to collect the behavioral response data. The reaction time was the lapse time from when the object appeared on the screen when there was a correct response. An error was considered to be an incorrect response or no response at all. The number of incorrect responses and missed targets were divided by the number of total trials in a condition to calculate the error rate.

### Fatigue and Order Effect

Performance could be influenced by fatigue or a learning effect. To address these concerns, the participants were randomized into one of three possible starting points in the series of exercise conditions (Figure 1). Because an incongruent task must always follow a congruent task in order to establish an expectation of behavior, the randomization needed to be limited to these specific points. It is unlikely that cognitive fatigue played a role in this study, given the short duration of all activities and no increase in error rate in the final block of trials across all participants and conditions.

### Study Size

A power analysis (Erdfelder et al., 1996) indicated that a total sample of 24 participants would be needed to detect an effect size of 0.30 with 80% power using a repeated measure ANOVA. This would have a similar power from a previous study that tested the effects of acute exercise on cognitive performance with 2 x 2 mixed design used with an effect size of 0.31, with the power of 0.80 (Chang et al., 2014). Complete data from 32 total participants were obtained and analyzed to be conservative.

### Data Analysis

Data were visually reviewed, audited, and then scrutinized using descriptive statistics to confirm normality. First, each condition was compared using analysis of variance (ANOVA) with repeated measures. Second, the average step counts and HR as physiological responses were analyzed using a one-factor (Exercise Intensity) ANOVA with repeated measures. Also, each condition was compared using a paired-samples *t*-test to interpret the higher-order condition. Finally, behavioral responses of reaction time and error rate were analyzed using two-factor (Exercise Intensity × Cognitive Task) with repeated measures. When sphericity was violated in the ANOVA, the Greenhouse-Geisser correction was applied. *Post hoc* tests using the Bonferroni correction were performed to determine the location of significance. Differences were considered significant at *p* <0.05. When a difference reached significance, the effect size was calculated *via* Cohen's *d* or partial eta-square (${\eta_{p}}^{2}$).

## Results

### Participants

Of the 32 participants, a total of 29 participants completed the experimental sessions. Three participants did not complete the study. Two individuals had scheduled commitments and had to leave before the assessments were completed, and a third participant withdrew because of the perceived difficulty of testing conditions. Thus, all tasks were completed, and there were no missing trials with the 29 participants, whose data were included in the final analysis.

### Effect of Exercise Intensity During Dual-Tasking Conditions on Physiological Responses

**Figure 3:**
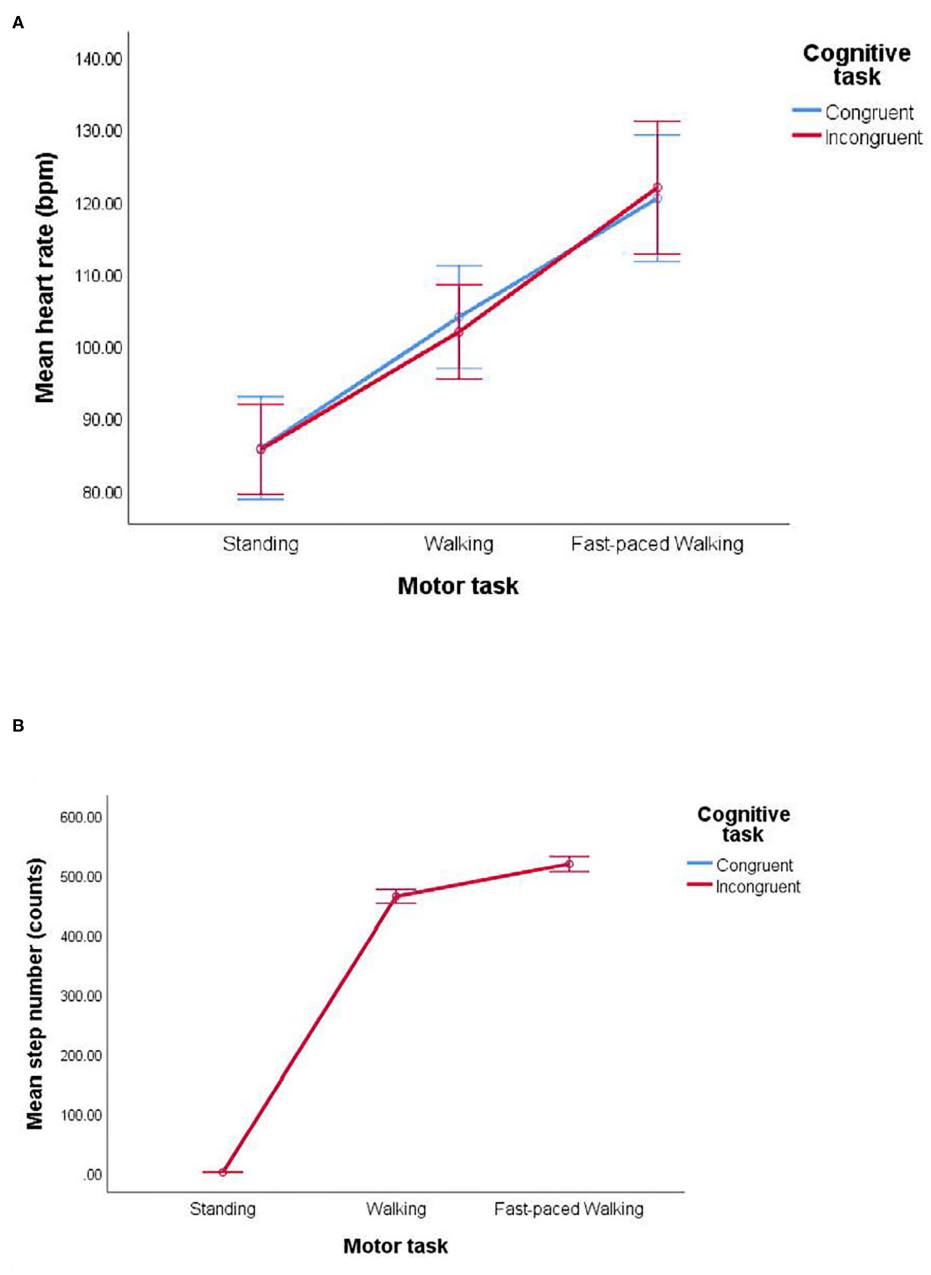
Physiological responses of **(A)** heart rates and **(B)** step counts during VR dual-tasking conditions.

The means of HR and step counts between conditions are presented in Table 2. The standing condition was excluded from this analysis because there was no significant variation in the number of steps detected across the participants. Instead, descriptive statistics were reported for the reference of full dual-tasking conditions (standing *M* HR = 85.71, *SD* = 17.35; standing *M* step counts = 0.60, *SD* = 1.15). A paired-samples *t*-test was used to determine whether there was a significant mean difference between walking and fast-paced walking conditions. There was a significant difference between dual-tasking conditions at each same gait speed on HR (walking: *M* = 102.93, *SD* = 17.79, fast-paced walking: *M* = 121.12, *SD* = 23.47, respectively; *SE* = 2.18, *t*(28) = 8.33, *p* < 0.01, *d* = 1.55, Figure 3) and step counts (walking: *M* = 462.86, *SD* = 29.89, fast-paced walking: *M* = 517.81, *SD* = 33.23, respectively; *SE* = 3.82, *t*(28) = 14.38, *p* < 0.01, *d* = 2.67). Although step counts for standing conditions should be “zero,” the sensors did detect a small motion artifact as participants moved over their bodies while standing, so some data shown were not exactly “zero.” Following the ActiLife software, the motion artifact does not change the movement classification of “zero” steps.

**Table 2:** Physiological and Behavioral Measures.

**Physiological Variables**	**Standing M (SD)**	**Walking M (SD)**	**Fast-paced walking M (SD)**	***p*-value**
HR, beats/min	85.71 (17.35)	102.93 (17.79)	121.12 (23.47)	<0.01**
Step counts	0.60 (1.15)	463.68 (30.10)	518.07 (33.81)	<0.01**
**Behavioral Variables**	**Standing M (SD)**	**Walking M (SD)**	**Fast-paced walking M (SD)**	***p*****-value**
Reaction time (sec)	0.63 (0.05)	0.58 (0.04)	0.59 (0.05)	<0.01**
Error rate (%)	13.11 (9.07)	2.84 (3.51)	4.06 (4.70)	<0.01**
**Behavioral Variables**		**Congruent M (SD)**	**Incongruent M (SD)**	***p*****-value**
Reaction time (sec)		0.59 (0.04)	0.62 (0.05)	<0.01**
Error rate (%)		7.30 (4.86)	6.03 (4.99)	0.18

Values are mean (SD). Significance difference by Bonferroni post hoc analysis, **p < 0.01: standing vs. walking, **p < 0.01: standing vs. fast-paced walking, **p < 0.01: walking vs. fast-paced walking.

### Effect of Dual-Tasking Conditions on Behavioral Responses

The reaction time and error rate for both congruent and incongruent tasks during treadmill exercise are presented in Table 2. There were no outliers, as assessed by examination of studentized residuals for values higher than ±3. The reaction time and error rate were normally distributed, as assessed by Shapiro-Wilk's test of normality on the studentized residuals (*p* > 0.05). Mauchly's test of sphericity indicated that the assumption of sphericity was violated for the two-way interaction, χ^2^ (2) = 7.66, *p* = 0.02. Therefore, we reported significance for the *F* values after the Greenhouse-Geisser correction.

**Figure 4:**
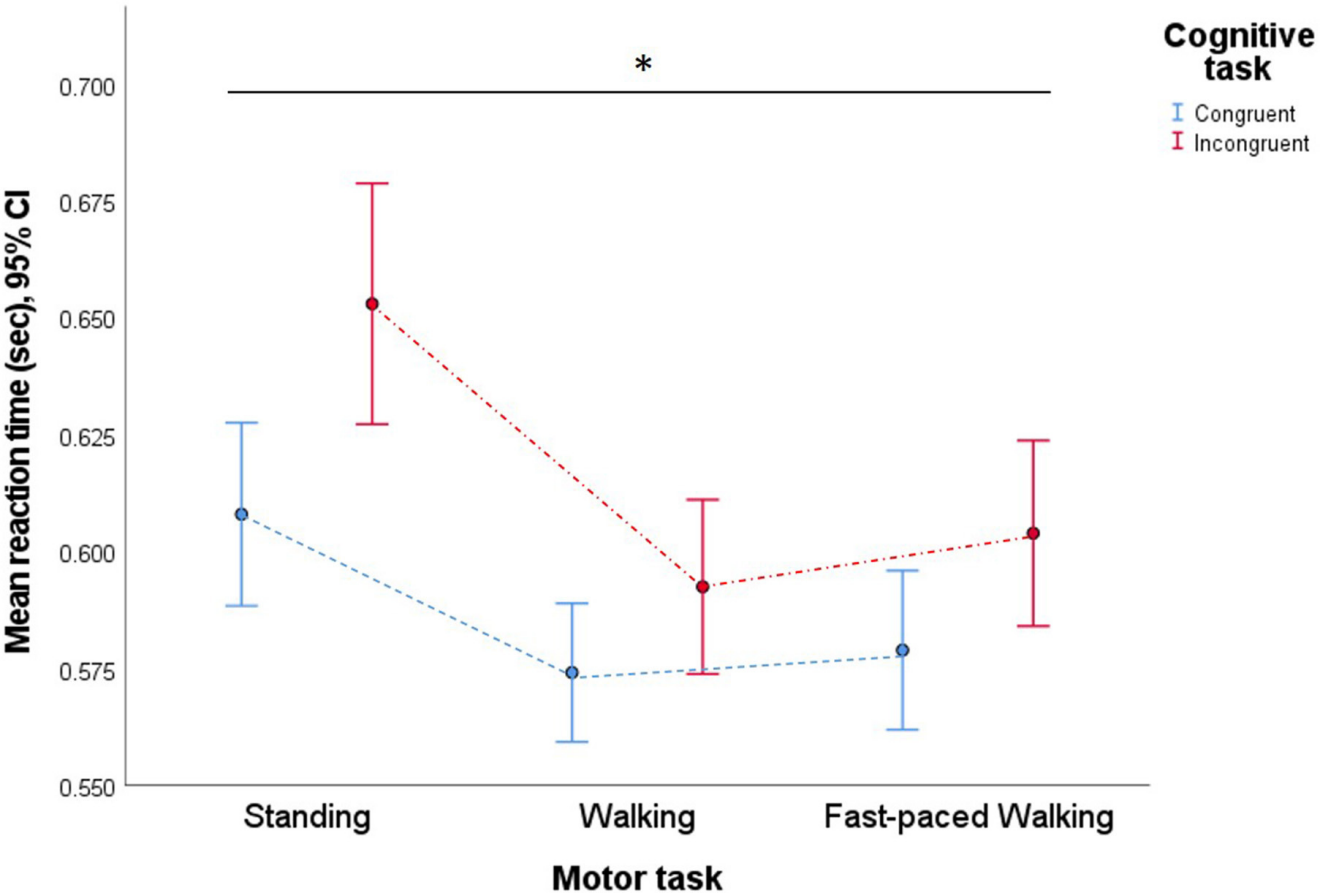
Means reaction time for the congruent and the incongruent task in all VR dual-tasking conditions. Figure showing two-way interaction between motor task and cognitive task on reaction time (*p < 0.05).

A two-way repeated measures ANOVA was calculated to determine the effect of each dual-tasking condition on reaction time. There was a significant two-way interaction between exercise intensity and cognitive task [*F*_(1.60, 44.91)_ = 3.56, *p* < 0.05, ${\eta_{p}}^{2}=$ 0.11] on reaction time (Figure 4). Therefore, simple main effects were run. First, a simple main effect for cognitive tasks for differences in reaction time between conditions at the same level of exercise intensity was run. Three separate tests were analyzed using one-way repeated measures ANOVA. Mean reaction time was 0.045 s, 95% CI [0.024–0.066] faster at the standing congruent task as opposed to the standing incongruent task, a significant difference, *F*_(1, 28)_ = 18.95, *p* < 0.01, ${\eta_{p}}^{2}\;=$ 0.40. Mean reaction time was 0.018 s, 95% CI [0.005–0.032] faster at the walking congruent task as opposed to the walking incongruent task, a significant difference, *F*_(1, 28)_ = 7.51, *p* = 0.011, ${\eta_{p}}^{2}=$ 0.21. Mean reaction time was 0.025 s, 95% CI [0.014–0.037] faster at the fast-walking congruent task as opposed to the fast-walking incongruent task, a significant difference, *F*_(1, 28)_ = 19.65, *p* < 0.01, ${\eta_{p}}^{2}\;=$ 0.41. *Post hoc* tests using the Bonferroni correction indicated that mean reaction time was significantly faster at the congruent task (*M* = 0.59, *SD* = 0.04) than the incongruent task (*M* = 0.62, *SD* = 0.05), *F*_(1, 28)_ = 34.31, *p* < 0.01, ${\eta_{p}}^{2}=$0.55, a difference of 0.03 s, 95%CI [0.02–0.04]. Second, a simple main effect for exercise intensities for differences in reaction time between conditions at each level of the same cognitive task was run. Two separate tests were analyzed using one-way repeated measures ANOVA. Mean reaction time was significantly changed over the different intensity of exercise in the congruent task, *F*_(2, 56)_ = 11.64, *p* < 0.01, ${\eta_{p}}^{2}\;=$ 0.29. There was also a significant effect of different intensity of exercise in the incongruent task, *F*_(2, 56)_ = 25.65, *p* < 0.01, ${\eta_{p}}^{2}=$0.48. *Post hoc* tests using the Bonferroni correction indicated that mean reaction time was significantly faster during the walking condition (*M* = 0.58, *SD* = 0.04), a difference of 0.05 s, 95% CI [0.03–0.06] and fast-paced walking condition (*M* = 0.59, *SD* = 0.05), a difference of 0.04 s, 95% CI [0.02–0.06] than standing condition (*M* = 0.63, *SD* = 0.05), *F*_(1.50, 42.02)_ = 30.18, *p* < 0.01, ${\eta_{p}}^{2}\;=$ 0.52. However, there was no difference between walking and fast-paced walking conditions (*p* = 0.32).

**Figure 5:**
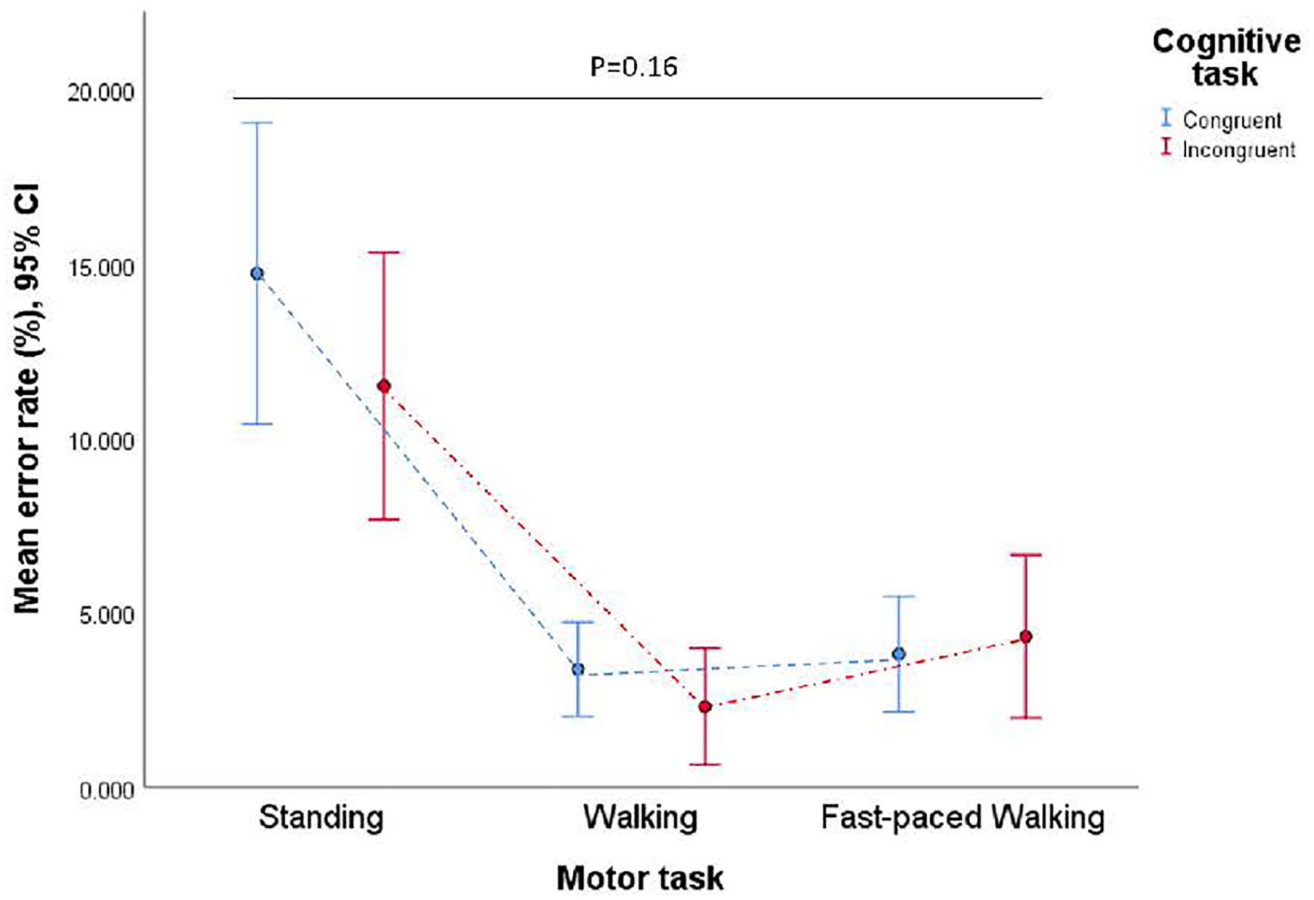
Means error rate for the congruent and the incongruent task in all VR dual-tasking conditions. Figure showing no interaction between motor task and cognitive task on error rate.

A second two-way repeated measures ANOVA was conducted to determine the effect of each dual-tasking condition on the error rate. There was no significant two-way interaction between exercise intensity and cognitive task [*F*_(1.30, 36.47)_ = 2.07, *p* = 0.16, ${\eta_{p}}^{2}\;=$ 0.07] on error rate (Figure 5). The main effect of exercise intensity showed a significant difference in error rate between conditions, *F*_(1.36, 38.20)_ = 29.33, *p* < 0.01, ${\eta_{p}}^{2}=$0.51. *Post hoc* tests using the Bonferroni correction indicated that mean error rate was significantly higher in the standing condition (*M* = 13.11, *SD* = 9.07) than walking condition (*M* = 2.84, *SD* = 3.51), a difference of 10.27 percentages, 95% CI [5.84–14.70] and fast-paced walking condition (*M* = 4.06, *SD* = 4.70), a difference of 9.05 percentages. However, the main effect of cognitive task showed that there was no significant difference in error rate between conditions, *F*_(1, 28)_ = 1.92, *p* = 0.18, ${\eta_{p}}^{2}=$ 0.06.

## Discussion

To the best of our knowledge this is the first dual-tasking study using the VR environment to measure congruent and incongruent tasks during exercise. Three different dual-tasking conditions were developed to offer varying exercise intensities and cognitive demands. Our findings indicated four main things. First, we confirmed that using VR is feasible and produces the physiological response that were anticipated. Second, the motor task with different intensities of exercise affects the behavioral response.

Contrary to our hypothesis, however, walking and fast-paced walking conditions on the VR treadmill led to significantly faster reaction time and a lower error rate than the standing condition. Third, the congruent task elicited faster reaction times but not lower error rates than the incongruent task. There was also an interaction effect on reaction time combined with the physical and cognitive demanding conditions but not on error rates. Finally, the VR treadmill has a unique potential to create VR conditions with various gait speeds and different levels of cognitive engagement.

### Feasibility of the VR Condition

The researchers intended to develop replicable conditions to examine the effects of exercise intensity and mode on cognitive performance. Once established, this would permit the examination of cognitive performance before, during, and after exercise. The study has value because a prominent health risk factor is physical inactivity or sedentary behaviors, which have risen steadily. Our primary objective was to determine the feasibility of the varying dual-task conditions using the VR treadmill. The researchers measured each individual's physiological cost to confirm the reliability to consistently produce the expected energy demands (Li et al., 2005; Schaefer, 2014). Li et al. (2005) had previously identified several limitations of dual-tasks assessments: (a) the laboratory environment should be as close as possible to real-world settings; (b) performance of each task and their interrelations should be compared for all tasks involved; and (c) task difficulties should be systematically varied to challenge individuals at appropriate levels. The researchers asked the participants to respond as fast as possible to a stimulus representing a visual distraction during the presented VR conditions parallel to real-world experience. Our study used a 180° degree VR projection screen and generated six different conditions with varying cognitive and motor difficulties. General dual-tasking studies typically used disconnected cognitive tasks. However, activities people perform in the real world far more commonly involve balancing tasks that integrate with their movements (e.g., using a cell phone while walking, etc.). The present study used the VR system to measure the cognitive task integrated with the participants' movement responses. Thus, this extends the literature regarding the measurement of combined physiological and behavioral effects to more ecologically relevant scenarios.

### Exercise Intensity Validation Through Physiological Responses

The majority of treadmill walking studies confirmed that speed was the most common reported gait outcome measure, reflecting its practical simplicity and clinical usefulness (Al-Yahya et al., 2011). This study used gait speed to control three different exercise intensities. This generated a significant difference between each condition on the HR and step counts. The researchers assumed that PWS and fast-paced walking conditions would generate light to moderate intensity of exercise and that this process could be verified through accelerometer measures because the use of steps/min data through accelerometry has become a promising method to validate exercise intensity categories (i.e., light, moderate, high) in young adults (Treuth et al., 2004). The researchers experienced some difficulties using the accelerometers to establish the thresholds for vigorous activity. However, the average step counts of the PWS walking condition (116.13 steps/minute), and fast-paced walking condition (129.38 steps/minute), did align with the light to moderate activity intensity categories established by Tudor-Locke et al. (2005).

Polar HR data was used to determine the intensity of exercise between conditions, because it is linearly related to oxygen uptake for dynamic activities (Freedson and Miller, 2000; Strath et al., 2002), which is the standard measure of energy expenditure during non-maximal or stress test treadmill studies. HR during standing condition was significantly higher than resting HR [*t*(28) = 9.93, *p* < 0.01], which implied that the standing condition itself (*M* = 85.71, *SD* = 17.35) has a short bout of light-intensity exercise with 25–40% heart rate reserve (HRR) by the method of Karvonen formula (Karvonen and Vuorimaa, 1988). Also, the PWS condition trained at 40–55% of HRR, between light and moderate-intensity exercise categories. Comparing step counts and HR indicated that PWS condition showed the light to moderate intensity of exercise, which might facilitate cognitive processing more than standing (light-level) and fast-paced walking (moderate-level) conditions under the dual-task paradigm. Given the different performance responses, our findings are supported by the inverted U-shape of arousal. Further, in pre/post designed experiments with individuals who participate in moderate to vigorous acute bouts of physical activity, reaction time is lower post-exercise than pre-exercise (Hogan et al., 2013). Although these effects are not as strong as those among older adults, they are still statistically significant and clinically relevant, as faster responders can process information more rapidly. Specific to dual-tasking contexts, physical activity could lead to enhanced processing when expedited reallocation of attentional resources is needed, perhaps even required for survival (e.g., avoid an oncoming car).

### Behavioral Response of Walking and Fast-Paced Walking

Given the known association between gait and cognition, the researchers hypothesized that performance would deteriorate when the participants walked. The hypothesis did not hold, and instead, the opposite occurred, with walking and fast-paced walking having better reaction time and error rate than the standing condition. Despite the starting point being randomly assigned, the increases in behavioral responses and cognitive performance were likely because these healthy young participants were at their cognitive peak and could shift their attention from one task to another without a detectable decline in performance. Further, performance could have improved because walking is a rote task, and attention was shifted to the cognitive task. Among 40 young active and sedentary adults (*M* age = 21.4 years of age), task-switching performance, measured as reaction time, was superior among those who were regularly active (Kamijo and Takeda, 2010). The male participants in this study were classified as healthy, while the females were classified as overweight. However, on the health questionnaire, 85% of the participants stated that they were meeting the national physical activity guidelines of 150 min of moderate to vigorous physical activity each week. Although beyond the scope of this study, health risk factors could have influenced the results.

Moreover, the cognitive tasks were based on the Go/No-Go paradigm and likely were too simple for the educated participants, and therefore what they experienced was an environment of mutual facilitation (Plummer et al., 2013). In this environment, all congruent/incongruent tasks provided both visual and auditory feedback, so when an error was made, the participant could learn from their mistake and adapt their performance. Since there was no significant order effect among the conditions, the researchers concluded that the condition's integrity was preserved. The participants in this study have potentially experienced gaming and VR environments in daily living and overcame the potential for increased risk for variability associated with dual-task interference (Chopra et al., 2018) because of their ability to shift their attention from one stimulus to the next. Future studies should include VO_2_ max testing as an objective measure of fitness. Further, participants should be screened for previous VR and gaming experiences to account for possible historical factors that may have influenced internal validity.

### Behavioral Responses of Reaction Time and Error Rate

Previous dual-task studies that combined exercise with cognitive test conditions frequently report degraded cognitive performance, reflecting slower reaction time (Plummer and Eskes, 2015; Herold et al., 2018). However, this study revealed the facilitation of participants' cognitive performance during treadmill walking rather than standing.

Several possibilities can be interpreted from these results. First, treadmill walking is usually highly automatized and does not necessarily lead to performance decrements in cognitive processing (Lövdén et al., 2008; Schaefer et al., 2010). This result is supported by other dual-task studies suggesting that when the pace on the treadmill is preferred, it does not interfere with the attention demand of motor tasks (Bloem et al., 2001; Tomporowski and Audiffren, 2014). Further, treadmill walking did not include any perturbation, which can happen in real-life walking. Patel et al. (2014) investigated the effect of different cognitive tasks and gait speeds on cognitive-motor interference of dual-task walking in young adults. As they remarked, PWS on the treadmill can prioritize complex cognitive tasks requiring higher attentional and processing resources over walking. Rather than sitting and slow-speed walking, PWS walking showed the most effective way to perform the cognitive task under the dual-task structure.

Second, it is possible PWS walking itself might lead to a better performance of the cognitive task. Our study results support the previous studies that preferred walking gait speed during dual-tasking conditions has beneficial effects on cognitive performance (Beauchet et al., 2005; Yogev-Seligmann et al., 2010; Al-Yahya et al., 2011). The cognitive improvement during dual-task walking is consistent with the “inverted-U shape theory” for cognitive processing (Yerkes and Dodson, 1908; Anderson, 1990). The inverted-U theory assumes that when physical arousal increases, performance is predicted to improve up to a maximum point and then to deteriorate with further increases in physical arousal. For example, Kamijo et al. (2007) investigated the effect of exercise intensity and task difficulty on cognitive processing using the P3 component of event-related brain potential. The P3 amplitude increased across task conditions following light and moderate cycling, but not during hard cycling, relative to baseline. The authors concluded that P3 amplitude might change in an inverted U-shape fashion due to acute exercise intensity. This inverted-U theory suggests that the optimal arousal level might depend on the difficulty of the given task. More specifically, if a task is complex in a dual-task paradigm, moderate cognitive and physical arousal levels might result in better performance.

In contrast, high/low levels of arousal will result in a deterioration of performance. If, however, the task is simple, it might require higher levels of arousal for optimal performance to be exhibited (Kamijo et al., 2004; Pesce, 2012). Schaefer et al. (2010) reported similar results with a young adult age group that participants' working memory performance was facilitated when walking at the preferred speed, but not at a fixed slower pace. They concluded that the interaction of walking and cognitive performance is influenced by sharing resources under the dual-task paradigm, and an exercise-induced activation of resources may cause that performance improvement in the cognitive task. In the present experiment, participants' reaction times and error rates changed appreciably when they walked at their PWS, compared to standing. Even though fast-paced walking did not show a significant difference in performance, we believe this was because the treadmill speed was not fast enough to deteriorate the attentional resources.

### Adapted Congruent/Incongruent Task Comparison Under the Dual-Task Paradigm

Different results can be found in cognition-exercise studies depending on which cognitive task is applied in the dual-task paradigm. Our study utilized an adapted congruent/incongruent task of the Go/No-Go paradigm, which elicits inhibition control. Average scores of the congruent tasks during treadmill exercise showed faster reaction time than average incongruent tasks, but there was no difference in the error rate. Given that result, the incongruent task required more cognitive processing time than the congruent task, but there was no difference between the tests on the error rate. This finding suggests that they needed more time to plan the response movement during the incongruent task, but they could still execute those movements appropriately. McMorris and Hale (2012) and McMorris (2015) obtained a similar result in which processing speed may facilitate exercise, but not on the error rate factor. Accuracy improvement took place only when the timing of testing was post-exercise, not during exercise.

### Strengths and Limitations

The present study has several strengths and limitations. Among the strengths are an attempt to develop an ecologically valid VR condition for testing exercise dose-response effects on cognitive performance. Also, there was a direct comparison of performance by condition within each participant. Finally, the use of VR to create a realistic simulation of the natural conditions within a controlled lab setting was novel and opened the door for new ways to understand the relationship between exercise and cognitive performance. As for the limitations, first, participant's standing conditions may have been a disadvantage for the reaction time because the condition did not result in a natural arm swing. Although all stimuli were blocked and displayed for the same duration on the screen, future studies should address this limitation by using markers to measure work and distance from the standing point and stimuli. Second, dual-task interference or facilitation to support the inverted-U theory may depend on the cognitive test selected. Our study only used adapted congruent/incongruent tasks for the cognitive assessment, but future research should encompass several cognitive performance tasks with variable demands to better understand this relationship. Third, behavioral responses were classified and dichotomously coded as correct or incorrect, and as such, we do not have a full understanding of why a participant may not have elected to respond to a displayed stimulus.

## Conclusion

Motor-cognitive dual-tasking limited attentional resources in various settings. Our study confirmed that dual-task-related changes in gait speed are sensitive to the performance and could reflect the exercise intensity differences with HR and step counts responses. PWS condition generated lower reaction time and error rate than standing condition, which implicated that self-paced walking can increase blood flow in the prefrontal cortex. Cognitive demanding differentiation while treadmill exercises impact the reaction time, but not the error rate within these conditions. This result implied that speed of processing during congruent task while the VR treadmill condition was faster than incongruent task while VR treadmill condition, but not on the cognitive performance accuracy. Future studies need to identify the brain mechanisms underlying arousal-induced resource activation. Therefore, physiological data (e.g., blood draw) or brain data (e.g., fNIR) can incorporate with advanced technology such as the VR treadmill. Although the present study only investigated young adults' age groups, children and older adults, or humans acquiring a new motor skill might be a relevant group comparison study.

## Data Availability Statement

The raw data supporting the conclusions of this article will be made available by the authors, without undue reservation, to any qualified researcher.

## Ethics Statement

The studies involving human participants were reviewed and approved by University of Texas at Austin. The participants provided their written informed consent to participate in this study.

## Author Contributions

YJ: conceptualization, planning, data collection, data analysis, and writing of the manuscript. JD: conceptualization, planning, writing of the manuscript, supporting proofreading, and supporting data analysis. PC: protocol development, data collection, and data analysis. BB: data collection, data analysis, and writing of the manuscript. DC: conceptualization, planning, and supporting proofreading. All authors: contributed to the article and approved the submitted version.

## Conflict of Interest

The authors declare that the research was conducted in the absence of any commercial or financial relationships that could be construed as a potential conflict of interest.
